# Requirements for improving social capital among faculty members of medical universities: A multicenter qualitative study

**DOI:** 10.1002/hsr2.1113

**Published:** 2023-02-13

**Authors:** Mohammad Hasan Keshavarzi, Saeed Shahabi, Ahmad Kalateh Sadati, Maryam Hashempour‐Sadeghian, Leila Zarei, Ali Ardekani, Ali Akbary, Mohammadreza Zakeri, Seyed Taghi Heydari, Kamran Bagheri Lankarani

**Affiliations:** ^1^ Clinical Education Research Center, School of Medicine Shiraz University of Medical Sciences Shiraz Iran; ^2^ Health Policy Research Center, Institute of Health Shiraz University of Medical Sciences Shiraz Iran; ^3^ Department of Social Sciences Yazd University Yazd Iran; ^4^ Faculty of Economics, Management and Social Sciences Shiraz University Shiraz Iran; ^5^ Department of Psychiatry, Faculty of Medicine, Social Development & Health Promotion Research Centre Gonabad University of Medical Sciences Gonabad Iran

**Keywords:** faculty members, medical education, medical schools, organizational identification, social capital

## Abstract

**Introduction:**

Social capital is critical to organizational dynamics, particularly in developing countries. This study explored strategies for enhancing social capital among faculty members at seven medical universities in the south of Iran.

**Methods:**

This qualitative study was conducted in 2021. We used a purposeful sampling technique to recruit faculty members and conducted individual semi‐structured interviews with them. Thematic analysis was used to analyze and describe the collected data.

**Results:**

A total of 49 faculty members (34 males; 15 females) participated in this study. The participants expressed satisfaction with their affiliations with medical universities. Social capital was related to the feeling of belonging to the organization, as well as to interpersonal and intra‐organizational relations. Social capital was associated with three components: empowerment, organizational policy change, and organizational identification. Additionally, a dynamic relationship between the individual, interpersonal, and macro‐organizational levels reinforced the organization's social capital. This means that, just as the macro‐organizational level affects the members' identities, the members' activism affects the macro‐organizational level.

**Conclusion:**

To strengthen the organization's social capital, managers should work on the mentioned components at the individual, interpersonal, and macro‐organizational levels.

## 1 INTRODUCTION

Medical schools require qualified and competent faculty members to achieve their educational objectives.^1^ Faculty members play the most critical role in improving the quality of educational delivery as they are the primary constituents of universities.^2^ Thus, special attention must be paid to these critical and valuable human resources, and strategies for faculty member growth and empowerment must be developed.^3^, ^4^

Faculty member development is defined as assisting faculty members in improving their skills, developing more effective curricula, and improving the organizational environment for teaching.^5^ One aspect that can be utilized as a part of a faculty member development program is improving social capital. Social capital is a term that refers to the characteristics of a social organization, such as networks, norms, and trust, that emerge from the organization's atmosphere and facilitate coordination and cooperation among its members in pursuit of the common good.^6^ Social capital plays a much more significant role in organizations and societies than other capitals, and mass and group relation networks integrate people's relationships with organizations. Without social capital, other capitals become ineffective, and cultural and economic development paths become uneven and complex.^7^

Several studies have established the effects of social capital on various components of organizational behavior. A study reported a significant positive relationship between social capital and happiness among faculty members.^8^ Enhancing social capital in academic environments has significant benefits and positively affects the entrepreneurial behaviors of university faculty members. Rafiei et al. investigated the relationship between social capital and entrepreneurial behavior among faculty members. The findings indicated that these two variables are positively correlated.^9^ Social capital not only affects the performance of the faculty member but can also affect the performance of medical students.^10^ In fact, a recent study reported that underperformance by ethnic minority students might be justified through lower levels of social capital.^11^ Thus, managers should build social capital within their organizations to foster professional and entrepreneurial performance as well as academic achievements.

To the best of our knowledge, no study has been conducted to determine the factors affecting the social capital of faculty members of medical schools. This qualitative research assessed the most significant obstacles and solutions for increasing the social capital of this target population.

## 2 METHODS

This qualitative study was conducted at seven medical universities (Shiraz, Hormozgan, Bushehr, Yasuj, Jahrom, Larestan, and Fasa universities of medical sciences, referred to as “district five universities”) in the south of Iran (links to websites for further details are available in Supporting Information: Table S1). Semistructured interviews were conducted with faculty members of these universities. The protocol for this study was reviewed and approved by the Iranian National Center for Strategic Research in Medical Education.

### 2.1 Sampling strategy

Purposive sampling was used to select the participants. The sampling procedure was designed to collect participants who were as diverse as possible in terms of gender, university, academic rank, academic field, and experience. Additionally, high‐potential faculty members were identified using the Iranian Scientometric Information Database (https://isid.research.ac.ir/). The participants were included if they were employed full‐time at one of the mentioned universities and had a minimum of 3 years of work experience. Sampling was carried out until data saturation was achieved. Two research team members independently compared the four most recent interviews with previous interviews to ensure data saturation.

### 2.2 Data collection

Individual semistructured interviews were conducted face‐to‐face or via telephone after obtaining the participant's consent. No third parties were present during the interview sessions, and the interviewee and interviewer conversed in a quiet environment. Each participant received an email with a detailed explanation of the research and the interviewer's identity before the interview. Furthermore, the interviewer repeated the study objectives orally at the start of each interview. The interview sessions were guided and facilitated by a predefined protocol. We utilized a literature search to determine the first draft of questions, and a consensus between the authors was reached subsequently. The interview questions were revised in response to feedback from the initial sessions. Among the questions addressed in this guide were: (1) What factors influence faculty members' social capital? (2) How can faculty members' social capital be strengthened? (3) Which strategies have been employed by other countries? If the interviewee consented, the interview was recorded; otherwise, the interviewer took notes for later analysis. Following each interview, the transcript was written.

### 2.3 Data analysis

Data analysis was conducted concurrently with the interviews. The collected data were analyzed using Braun and Clarke's thematic analysis approach.^12^ The texts were first reviewed repeatedly by two research team members, after which the initial codes were extracted. The relationships between the extracted codes were then determined, and subthemes were assigned. Finally, the obtained subthemes were examined, and closely related items were classified into major themes. Any disagreements between the authors were resolved via discussion, and a third author was consulted if necessary. The authors who participated in this phase came from various executive and scientific backgrounds to mitigate potential bias. Moreover, critical reflexivity was considered during data analysis to minimize the possibility of bias. Data were analyzed using the MAXQDA 10 software (VERBI GmbH). The data collection and analysis processes included credibility, transferability, confirmability, authenticity, and dependability to ensure the findings were trustworthy.^13^ To meet these criteria, the primary investigator was involved throughout the research, the collected data were verified with the participants, the authors checked each other's work, and the sample was selected with the maximum possible variety. Additionally, the standards for qualitative research reporting (SRQR)^14^ guidelines were used to improve the reporting and methodological quality.

## 3 RESULTS

We interviewed 49 individuals, including 34 male and 15 female faculty members. The characteristics of the participants are listed in Supporting Information: Table S2. Following data analysis, three major themes and eleven subthemes were identified (Table 1). The sections that follow summarize the study's most significant findings.

**Table 1:** Themes, subthemes, and codes.

Theme	Subtheme	Code
Empowerment	Interpersonal communication skills	Mutual respect How to negotiate
	Conflict resolution	Ability to resolve conflict Adaptability Problem‐solving
	Teamwork	Collective interest Group solidarity Synergy
Changes in organizational policies	Revising the rules	Accepting ideas Understanding people's needs
	Improving trust	Transparency Respect for the rules
	Supporting collective activities	Participation in projects Rewarding participatory activities
	Collaborative planning	Participate in decision making Identify talents
Organizational identification	Appreciation	Appreciation of faculty members Mutual respect
	Sense of group identity	Membership Sense of worth Empathy Sense of impact Emphasis on values
	A culture of accountability	Mutual commitment Work conscience Responsibility
	Fostering a common discourse	Knowledge sharing Participation in organizational knowledge

### 3.1 Empowerment

Empowerment is defined as a collection of training and skills that pave the way for professional development in the organization's collective and participatory activities.

#### 3.1.1 Interpersonal communication skills

Interpersonal communication skills entail communicating with others and developing relationships to foster greater understanding and interaction. One participant stated: “We need to teach effective communication skills and how to interact in the organization. The role of interactions in the organization and its consequences in individual and organizational success should be taught” (No. 8). Another participant mentioned, “How we interact with each other in the organization is a treasured part of job satisfaction; the effectiveness of the organization is summed up in the way we interact” (No. 31).

#### 3.1.2 Conflict resolution

Conflict resolution includes techniques for managing and resolving interpersonal conflicts and clashes. One of the participants stated: “They should train the staff on how to solve their problems in the university hospital environment and how to get along, how to solve the problem, and how to hear opposing ideas and use them to benefit the organization.” (No. 5). “Over the years, I have seen several research projects that have failed due to divisiveness and hostility [between colleagues], stemming from a lack of mutual understanding” (No. 18).

“What we need to teach is to have diversity among faculty members in the sense that we can bring together professors with different tastes and abilities in the areas of performance, research, education, and treatment” (No. 12).

#### 3.1.3 Teamwork

Teamwork is defined as a specific way of working that aims to maximize the effectiveness of group abilities and skills. One of the participants said: “Organ transplantation is highly complex, and expectations have risen, so the only solution is to use teamwork to increase the accuracy and quality of work. You are now witnessing how a thousand people must perform specialized tasks to send one person to conduct space research. Previously, such issues did not exist” (No. 22).

“Once upon a time, there was only a simple phone. Now look at the smartphone with such complexity and the variety of software available on the market … don't you think a team has designed and created it?” (No. 44).

“The university is not isolated from society. In the past and since childhood, we were raised with individualism. Let us look back and see why today we behave individualistically. Cartoons, movies, stories, and teachers' encouragement methods were all based on individualism. How should we behave in the opposite direction? … Perhaps this is why we achieve more success in individual sports than in team sports” (No. 17).

### 3.2 Changes in organizational policies

Changes in organization policies refer to the abolition, addition, or modification of existing organizational laws and regulations to expand and develop the organization's social capital.

#### 3.2.1 Revising the rules

Revising the rules refers to the desire to eliminate some regulatory barriers and replace them with more compelling ones consistent with the organization's ambitious goals. One of the participants stated: “The rules that are set for academic promotion make the individual only think about upgrading their own status; the rules should be directed toward collaboration and teamwork. For example, if you earn one point from publishing an article per promotion regulations, there should be more points for articles that multiple authors write. That is, we should push faculty members towards teamwork” (No. 23).

“As long as the board of trustees makes decisions in universities and has the authority to act contrary to the Ministry's instructions, nothing will change. The Ministry has approved several good regulations, but the university does not implement them” (No. 28).

“The university administration should define a new policy to promote inter‐university synergy” (No. 2),

“The current rules need to change. If they do, valuations will change as well. Someone who works part‐time and spends most of their time outside the university is valued as a smart person … A person who spends their entire time at university and continues to work here is referred to as inept” (No. 9).

#### 3.2.2 Improving trust

Improving trust is generally understood to mean assuring the organization's employees that its programs and objectives align with what officials do. “Faculty members need to be respected. University administrators and policymakers need to understand their needs and wants. They need to know the concerns of their colleagues. That is when trust is built,” said participant No. 35.

“In any case, I do not think that slogans are enough to influence the employees. The solution is that the organization should prove that it wants the promotion and progress of everyone within the organization. That is, people should feel trust in their organization … in their colleagues … and in the laws of their country.” (No. 8)

#### 3.2.3 Supporting collective activities

Supporting collective activities is any action the organization takes to support employees' team and group activities. One participant stated: “At my university, I am told that I should write single‐author articles, whereas multiauthor papers are valued at other universities” (No. 20).

“Our encouragement at the university should be toward group encouragement. While rewarding the group's leader is appropriate, the entire team should be appreciated and motivated, as each member contributed to the group's success or failure.” (No. 12)

#### 3.2.4 Collaborative planning

Collaborative planning embodies how candidates for senior management positions or key positions within an organization are chosen and recruited from a pool of qualified candidates. One of the participants said: “We should stop individuals from making decisions based on their personal view on behalf of everyone, as such actions will harm the university. We should assign responsibility to individuals in turn in all positions, similar to the 2‐year rotating terms of faculty members for the scientific department head position so that all individuals have an equal opportunity for promotion” (No. 21).

“Everyone should be given educational, research, and executive responsibilities at the university so they can push themselves and take on a role in the organization's management. This has several advantages. As I have managerial responsibilities in the hospital, I am familiar with the hospital's problems. I have experienced various challenges and have gained a better understanding of the organization's situation. I may also have ideas that can help the whole organization” (No. 10).

### 3.3 Organizational identification

The organization identification theme refers to the process of establishing a culture and a forum for academic discourse and inspirational motivation at the university.

#### 3.3.1 Appreciation

Appreciation is defined as an awareness of the value of the services provided by employees who have contributed to the organization's growth and development. In this regard, one of the participants stated: “Committed, diligent, experienced and exemplary professors should be praised … on different occasions like Pharmacists Day, Physicians Day, Nurses Day, and under various pretexts; it gives us a chance to value these people and honor their work” (No. 16).

“One solution is to have a coherent annual program to honor elite professors, as such role models can pass on their valuable experiences to the university's next generations through reflective sessions” (No. 26).

“The dignity of faculty members must be upheld” (No. 33).

“Leaders are very influential in the organization. Hierarchy, respect, and moral values must be demanded” (No. 21).

#### 3.3.2 Sense of group identity

A sense of group identity broadly refers to a person's sense of belonging to their place of work within the organization. If they have this sense, individuals desire to work for and grow with the organization. One of the participants said: “Professors should feel that the university has done something for them or contributed to their progress. Whatever memories they have of the university are associated with hope and companionship, which binds them to the institution, so that even if they go to study abroad for a period, they wish to return to their own country despite the attractiveness of the foreign universities” (No. 2).

“I must feel that the university is supporting me. I should feel important to the university. I must feel that the university is proud of me. I must feel that the university acknowledges my concerns” (No. 15).

“As a solution, all faculty members should feel involved in the university planning process. That is, if the university is devising a plan or making a decision, they should use everyone's opinions. From top to bottom, everyone's opinions should be considered” (No. 17).

“If I believe that the university administration is concerned about my life and well‐being, I will not neglect the university's progress. I will give the university my heart and soul if it provides adequate compensation, facilities, and amenities for myself and my family” (No. 29).

#### 3.3.3 A culture of accountability

One of the participants stated: “Many problems and challenges will undoubtedly disappear if everyone in the country is accountable for the task they perform and the responsibility they have taken. Accountability of each person in the organization effectively provides transparency” (No. 17).

#### 3.3.4 Fostering a common discourse

Fostering a common discourse refers to the mutual understanding of common knowledge, beliefs, and hypotheses among an organization's employees, achieved through dialog.

One participant said: “We should try to keep the university away from social indifference and isolation and the lack of dialogue between faculty members” (No. 11).

“For example, for the departments of basic medical sciences, we can have meetings about exam validity and reliability, examination standards, rules, student issues, and differences between faculty members. We did this in the hospital and at the university, and we succeeded. We should have similar meetings to share experiences” (No. 19).

“Unfortunately, the current policy is to have faculty members who mind their own business. Do not criticize! Do not talk! Do not disagree! In such a situation, you cannot expect teamwork, innovation, and growth” (No. 31).

## 4 DISCUSSION

**Figure 1:**
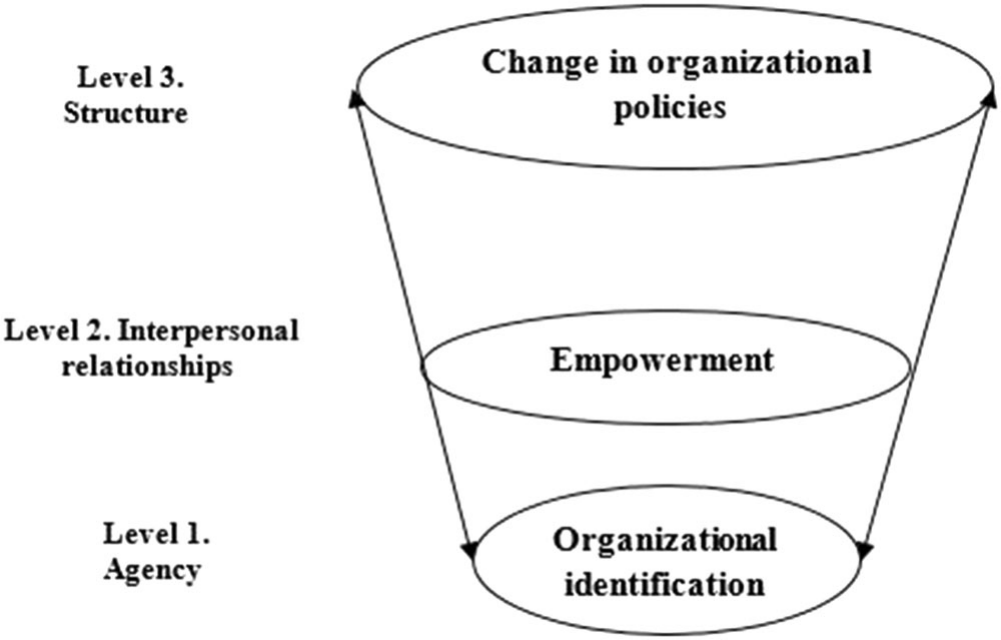
A schema of influential factors on social capital.

This study aimed to examine the development of social capital among medical faculty members in Iran's southern region. The findings indicated that social capital in this context encompasses three distinct dimensions: empowerment, policy change, and organizational identification. According to the research findings, strengthening social capital begins at the individual level, progresses to interpersonal skills, and is finally reproduced and sustained at the organizational layers. A multidirectional relationship exists between these levels (Figure 1). Individuals at the organization level define themselves by their interdependence, appreciation, mutual commitments, and ability to foster a common dialog with the medical science organization. Indeed, at this level, members develop values and beliefs that guide and motivate their organization's behavior.

Hu et al. believed that individuals are motivated to act and make sense of their work according to their identities.^15^ Additionally, an individual's social capital is comprised of various dimensions of an individual's communication network (i.e., prestige, resourcefulness, and friendships), and organizational identification is mediated by the perceived attractiveness of the organizational identity.^16^

According to He and Brown, organizational identity can be classified into four broad categories: functionalist, social constructionist, psychodynamic, and postmodern.^17^ Our research primarily focused on the constructivism theory, in which social capital is created through the interaction between individuals and the organization, with interpersonal relationships serving as an intermediary (Figure 1). It has been demonstrated that a person's social capital affects organizational identification, both directly and indirectly, through the perceived attractiveness of the organizational identity.^18^ The proposed model's second level (Figure 1) refers to the organization's network of interpersonal relationships, which includes interpersonal communication, conflict resolution, and teamwork. This is consistent with previous research emphasizing the importance of networks in organizations.^19^, ^20^, ^21^, ^22^

Gender, specialty, academic level, and college variables affect social capital.^23^ Another study conducted in Iran demonstrated that social capital, psychological capital, and organizational climate influence faculty burnout.^24^ This indicates the critical role and function of social capital (besides other capitals) in the organization. As a result, senior leaders at higher education institutions should consider social capital a tool for creating and developing both international and national educational partnerships.^25^

Social capital is an important component of any organization, contributing to the academic community's sense of ownership and belonging.^26^ This study demonstrates the validity of the constructivism theory of organization and social capital. This means that the agency derives its identity from the organization's context, and the organization reinforces the agency's identity. Furthermore, organizational changes are contingent upon the organization's employees and networks. As a result, each organization has unique dynamics influenced by its context and networks. Network dynamics alter corporate practices and the employment relationship between employees and employers.^23^, ^27^ Moreover, teaching‐focused social networks^19^ are recommended.

The present study indicates that leaders and managers of medical universities should take social capital very seriously and improve the social capital of their faculty members by fostering mutual trust, positive attitudes, and healthy social relationships between the organization's members and by appropriately distributing managerial roles and responsibilities among faculty members through careful macroplanning. As a participatory culture contributes significantly to the organization's sense of belonging and trust, improving relationships within the organization and creating a participatory atmosphere are also recommended.

The strengths of the present study include its multicenter nature, qualitative design, and robust methodology in investigating challenges and solutions for promoting social capital among faculty members of medical universities. However, the current study is limited in scope; therefore, generalizing the results to other countries should be accompanied with caution. On the other hand, the current study did not adequately address the network of relationships between organization members, including formal and informal relationships. Future research should examine the link between the organizational network and social capital more closely. Additionally, organizations in the 21st century appear to have further unknown and complex dimensions, necessitating a focus on postmodern perspectives in this area. Finally, while we focused merely on the network between faculty members, the relationship between faculty members and students should also be examined in future works as it is a component of the organization's social capital.^28^

## 5 CONCLUSION

The findings of this study indicate that the following strategies should be adopted to improve social capital among faculty members: (i) empowerment of the faculty members for professional development; (ii) consideration of social capital in organizational policies; and (iii) promotion of organizational identification through academic discourse and motivation at the individual, interpersonal, and macro‐organizational levels.

## AUTHOR CONTRIBUTIONS

**Mohammad Hasan Keshavarzi**: Conceptualization; Data curation; Formal analysis; Funding acquisition; Investigation; Methodology; Project administration; Validation; Writing – original draft; Writing – review & editing. **Saeed Shahabi**: Data curation; Formal analysis; Investigation; Software; Writing – original draft. **Ahmad Kalateh Sadati**: Data curation; Formal analysis; Investigation; Methodology; Validation; Writing – original draft. **Maryam Hashempour‐Sadeghian**: Data curation; Methodology; Writing – original draft; Writing – review & editing. **Leila Zarei**: Data curation; Formal analysis; Methodology; Writing – original draft. **Ali Ardekani**: Data curation; Writing – original draft; Writing – review & editing. **Ali Akbary**: Data curation; Formal analysis; Investigation; Writing – review & editing. **Mohammadreza Zakeri**: Data curation; Formal analysis; Investigation; Methodology; Writing – original draft. **Seyed Taghi Heydari**: Conceptualization; Data curation; Formal analysis; Funding acquisition; Investigation; Methodology; Project administration; Validation; Writing – original draft; Writing – review & editing. **Kamran Bagheri Lankarani**: Conceptualization; Project administration; Validation; Writing – original draft; Writing – review & editing. All authors read and approved the final version of the manuscript.

## CONFLICT OF INTEREST STATEMENT

The authors declare no conflict of interest.

## ETHICS STATEMENT

This study was approved by the Ethics Committee of the Iranian National Center for Strategic Research in Medical Education. (Code: IR. NASRME. REC.1400.296) Informed consent was obtained from all participants. Also, the authors confirm that all methods were carried out in accordance with relevant guidelines and regulations.

## TRANSPARENCY STATEMENT

The lead author Seyed Taghi Heydari affirms that this manuscript is an honest, accurate, and transparent account of the study being reported; that no important aspects of the study have been omitted; and that any discrepancies from the study as planned (and, if relevant, registered) have been explained.

## Supporting information

Supplementary information.

## Data Availability

The datasets used and/or analyzed during the current study are available from the corresponding author upon reasonable request.
