# Supplementary material for: Requirements for improving social capital among faculty members of medical universities: A multicenter qualitative study
Source: Health Sci Rep. 2023 Feb 13;6(2):e1113. doi: 10.1002/hsr2.1113 (PMC9925715; doi:10.1002/hsr2.1113)
Supplement: Supplementary file 1 — Supplementary information. [file HSR2-6-e1113-s001.docx]

| **Table S1. University websites for further details.** | |
| --- | --- |
| University websites | URLs |
| Shiraz University of Medical Sciences | <http://www.gsia.sums.ac.ir/en> |
| Hormozgan University of Medical Sciences | <https://en.hums.ac.ir/> |
| Bushehr University of Medical Sciences | <https://www.bpums.ac.ir/en/default.aspx> |
| Yasuj University of Medical Sciences | <https://en.yums.ac.ir/> |
| Jahrom University of Medical Sciences | <https://en.jums.ac.ir/> |
| Fasa University of Medical Sciences | <http://en.fums.ac.ir/> |
| Larestan University of Medical Sciences | <https://en.larums.ac.ir/> |

| **Table S2. Characteristics of the participants** | | | | |
| --- | --- | --- | --- | --- |
| **ID** | **Gender** | **Academic rank** | **Academic field** | **University** |
| **P01** | Male | Professor | Cardiology | Shiraz University of Medical Sciences |
| **P02** | Female | Professor | Community Medicine | Shiraz University of Medical Sciences |
| **P03** | Male | Assistant Professor | Emergency Medicine | Shiraz University of Medical Sciences |
| **P04** | Male | Assistant Professor | Internal Medicine | Shiraz University of Medical Sciences |
| **P05** | Male | Professor | Infectious Medicine | Shiraz University of Medical Sciences |
| **P06** | Male | Assistant Professor | Emergency Medicine | Shiraz University of Medical Sciences |
| **P07** | Female | Assistant Professor | Medical Education | Shiraz University of Medical Sciences |
| **P08** | Male | Assistant Professor | Surgery | Shiraz University of Medical Sciences |
| **P09** | Male | Associate Professor | Surgery | Shiraz University of Medical Sciences |
| **P10** | Male | Assistant Professor | Traditional medicine | Shiraz University of Medical Sciences |
| **P11** | Male | Associate Professor | Pharmacology | Shiraz University of Medical Sciences |
| **P12** | Female | Associate Professor | Pharmacology | Shiraz University of Medical Sciences |
| **P13** | Male | Professor | Ophthalmology | Shiraz University of Medical Sciences |
| **P14** | Male | Associate Professor | Psychology | Shiraz University of Medical Sciences |
| **P15** | Female | Assistant Professor | Dentistry | Shiraz University of Medical Sciences |
| **P16** | Male | Associate Professor | Dentistry | Shiraz University of Medical Sciences |
| **P17** | Male | Associate Professor | Oncology | Shiraz University of Medical Sciences |
| **P18** | Male | Professor | Internal Medicine | Shiraz University of Medical Sciences |
| **P19** | Male | Professor | Pharmacognosy | Shiraz University of Medical Sciences |
| **P20** | Male | Professor | Surgery | Shiraz University of Medical Sciences |
| **P21** | Male | Professor | Medical virology | Shiraz University of Medical Sciences |
| **P22** | Male | Professor | Medical Biotechnology | Shiraz University of Medical Sciences |
| **P23** | Female | Professor | Clinical Biochemistry | Shiraz University of Medical Sciences |
| **P24** | Male | Assistant Professor | Medical Education | Yasuj University of Medical Sciences |
| **P25** | Male | Associate Professor | Anatomical Sciences | Yasuj University of Medical Sciences |
| **P26** | Female | Associate Professor | Pregnancy Health | Yasuj University of Medical Sciences |
| **P27** | Male | Assistant Professor | Medical Immunology | Yasuj University of Medical Sciences |
| **P28** | Male | Assistant Professor | Pediatric Cardiology | Yasuj University of Medical Sciences |
| **P29** | Male | Assistant Professor | Pathology | Yasuj University of Medical Sciences |
| **P30** | Male | Professor | Bacteriology | Bushehr University of Medical Sciences |
| **P31** | Male | Assistant Professor | Environmental Health Engineer | Bushehr University of Medical Sciences |
| **P32** | Male | Professor | Pharmaceutical biochemistry | Bushehr University of Medical Sciences |
| **P33** | Female | Professor | Nursing | Bushehr University of Medical Sciences |
| **P34** | Male | Associate Professor | Infectious disease | Hormozgan University of Medical Sciences |
| **P35** | Female | Assistant Professor | Reproductive Biology | Hormozgan University of Medical Sciences |
| **P36** | Male | Associate Professor | Physiology | Hormozgan University of Medical Sciences |
| **P37** | Male | Associate Professor | Parasitology | Hormozgan University of Medical Sciences |
| **P38** | Female | Associate Professor | Pharmaceutical Nanotechnology | Hormozgan University of Medical Sciences |
| **P39** | Male | Associate Professor | Pharmacology | Fasa University of Medical Sciences |
| **P40** | Male | Associate Professor | Bacteriology | Fasa University of Medical Sciences |
| **P41** | Female | Assistant Professor | Medical Biotechnology | Fasa University of Medical Sciences |
| **P42** | Female | Assistant Professor | Medical Education | Fasa University of Medical Sciences |
| **P43** | Female | Associate Professor | Educational Planning | Jahrom University of Medical Sciences |
| **P44** | Male | Assistant Professor | Clinical Biochemistry | Jahrom University of Medical Sciences |
| **P45** | Associate Professor | Associate Professor | Nursing | Jahrom University of Medical Sciences |
| **P46** | Female | Assistant Professor | Tissue Engineering | Jahrom University of Medical Sciences |
| **P47** | Female | Assistant Professor | Pediatrics | Jahrom University of Medical Sciences |
| **P48** | Male | Assistant Professor | Emergency Medicine | Jahrom University of Medical Sciences |
| **P49** | Female | Assistant Professor | Nursing | Lar Medical School |
